# Use of nucleoside therapy for mitochondrial DNA depletion/deletion syndrome (MDDS)

**DOI:** 10.1016/j.neurot.2026.e01048

**Published:** 2026-08-18

**Authors:** Michio Hirano, Andres Berardo, Emanuele Barca, Valentina Emmanuele, Austin Larson, Antonella Spinazzola, Amel Karaa

**Affiliations:** a H. Houston Merritt Center, Department of Neurology, Columbia University Irving Medical Center, New York, NY, USA; b Hospital Allende, Department of Neurology, Universidad Católica de Córdoba, Córdoba, Argentina; c Clinical Genetics and Metabolism, Department of Pediatrics, University of Colorado Anschultz, Aurora, CO, USA; d Department of Clinical Neurosciences, University College London, London, UK; e Division of Clinical Genetics, Department of Pediatrics, Massachusetts General Hospital, Boston, MA, USA

**Keywords:** Nucleoside therapy, POLG, Thymidine, deoxycytidine, Thymidine kinase 2, TK2d

On November 3, 2025, the U.S. Food and Drug Administration (FDA) approved deoxycytidine/deoxythymidine (dC/dT) pyrimidine nucleoside therapy for clinical use in thymidine kinase 2 deficiency (TK2d), an ultra-rare autosomal recessive mitochondrial disease affecting patients with symptomatic onset at or before 12 years of age [1]. The European Commission subsequently granted marketing authorization on March 26, 2026 [2]. These approvals were based upon evidence from a pivotal phase 2 clinical trial, two retrospective multicenter chart reviews, and an expanded access program [3,4]. In matched-pair analyses of 82 treated and 93 untreated external control patients with disease onset at ≤12 years of age, nucleoside therapy was associated with a 92–94% reduction in the risk of death from time of treatment initiation [4]. Furthermore, among treated patients who had previously lost one or more motor milestones, 30 of 40 regained at least one milestone, including the ability to sit, stand, walk, or run [4]. These remarkable therapeutic advances have naturally stimulated interest in evaluating nucleoside therapy for other forms of mitochondrial DNA depletion/deletion syndrome (MDDS).

In this context, three exploratory studies have examined the use of dC/dT therapy in patients with non-TK2d MDDS, including POLG-related disease and other genetically heterogeneous disorders [[5], [6], [7]]. These reports include a 6-month interim analysis of an open-label study in POLG-related disease published in *eClinicalMedicine* [6], a heterogeneous MDDS cohort reported in the *Journal of Neurology* [5], and a longer-term follow-up study of the POLG cohort published in *Neurotherapeutics* [7]. Collectively, these investigations represent important early efforts to explore whether the clinical success observed in TK2d may extend to other disorders of mitochondrial DNA maintenance. Because those studies involve small, uncontrolled, and clinically heterogeneous cohorts, the findings should be interpreted as preliminary. In our view, the available evidence engenders several important questions regarding biological rationale, preclinical data, study design, and interpretation of efficacy that should be addressed as the field moves toward a definitive clinical evaluation.

## Scientific Rationale and Pre-clinical Evidence for dC/dT Treatment of MDDS

The development of nucleoside therapy for TK2d was founded on a well-defined mechanistic hypothesis supported by extensive preclinical investigation. Studies in both Tk2 knockin and knockout mouse models demonstrated tissue-specific imbalances in intramitochondrial deoxynucleoside triphosphate (dNTP) pools, characterized by deficiencies of deoxythymidine triphosphate (dTTP) alone or in combination with deoxycytidine triphosphate (dCTP). Oral administration of deoxycytidine monophosphate (dCMP)/deoxythymidine monophosphate (dTMP) or dC/dT ameliorated these biochemical abnormalities, delayed disease onset, and extended survival of TK2 knock-in mice by approximately two-to three-fold in a dose-dependent manner [8,9]. These findings established a strong biological rationale and preclinical evidence that ultimately supported clinical translation.

Whether a comparable mechanistic rationale exists for other MDDS disorders remains an important area of investigation. The studies evaluating dC/dT therapy in POLG-related disease acknowledge that the mechanism of action in these disorders “is not completely clear" [5,6]. To support the therapeutic rationale, the authors cite studies demonstrating accelerated recovery of mitochondrial DNA following ethidium bromide-induced depletion in POLG-mutant fibroblasts treated with a four-nucleoside combination (dC, dT, dA, and dG) in the presence of an adenosine deaminase inhibitor [10]. Although those experiments provide evidence that manipulation of nucleotide pools may influence mtDNA maintenance under experimental conditions, they did not assess pyrimidine nucleosides alone.

Direct evidence supporting the specific use of the two-nucleoside combination (dC/dT) in POLG disease is limited. To our knowledge, only one published study has evaluated dC/dT alone in POLG-mutant fibroblasts cultured under standard conditions. Although the investigators observed a trend toward increased cell numbers, the difference was not statistically significant, and no improvement in mitochondrial DNA copy number was demonstrated [11]. Consequently, while the available preclinical studies provide an initial rationale for further investigation, data from additional mechanistic studies and disease-specific preclinical models would substantially strengthen the biological foundation for extending dC/dT therapy beyond TK2d.

## Clinical Trial Design and Methodology

The clinical development of nucleoside therapy for TK2d illustrates the value of a stepwise translational approach integrating mechanistic studies, detailed natural history data, expanded access experience, and prospective clinical investigation. Following early expanded access protocols, clinical development transitioned to a prospective phase 2 study using pharmaceutical-grade Good Manufacturing Practice (GMP) dC/dT and regulatory-compliant data management [4,12]. In parallel, retrospective clinical data from expanded access programs, pretreatment cohorts, and published and unpublished cases were systematically assembled into a Good Clinical Practice (GCP)-compliant natural history database comprising 257 genetically confirmed TK2 patients. These efforts provided objective longitudinal data on survival and motor milestones without treatment that substantiated interpretation of treatment effects.

The recently published studies evaluating dC/dT therapy in non-TK2d MDDS disorders represent significant early efforts to explore whether the benefits observed in TK2d might extend to other disorders [[5], [6], [7]]. Given the rarity of these conditions, it is understandable that these investigations were conducted as open-label non-randomized studies involving relatively small patient cohorts at a single site. Such study designs are well-suited for evaluating feasibility, safety, and potential signals of biological activity. However, they also introduce significant limitations that must be considered when interpreting efficacy.

One key consideration is the marked clinical heterogeneity of POLG-related disease, which ranges from rapidly progressive childhood-onset Alpers-Huttenlocher syndrome to adult-onset chronic progressive external ophthalmoplegia, with intermediate phenotypes including ataxic neuropathy spectrum (ANS), myoclonic epilepsy myopathy sensory ataxia (MEMSA), isolated myopathy, and myocerebral hepatopathy syndrome (MCHS) [13]. Consequently, subjects enrolled within small exploratory studies may differ substantially in baseline disease severity, rates of progression, and concomitant symptomatic therapies. In the setting of such phenotypic diversity, it is challenging to recognize treatment-related effects.

Interpretation is further complicated by the absence of concomitant untreated comparator groups. Without an appropriate reference population, changes in functional outcomes or disease progression cannot readily be distinguished from the natural history of these disorders. Furthermore, the Newcastle Mitochondrial Disease Scale (NMDS), which is a validated clinical instrument of disease severity, is not an optimal outcome measure in non-blinded clinical trials because of its dependence on qualitative assessment, rendering it susceptible to expectation bias, observer bias, and scoring variability. In addition, in one participant, the six-month NMDS assessment was imputed from caregiver-reported information rather than direct clinical evaluation, illustrating the practical challenges of conducting non-industry standard clinical research in ultra-rare diseases [5].

Finally, statistical methodology deserves careful consideration. The published POLG studies employed one-tailed Wilcoxon testing [5,7]. This approach assumes that treatment effects occur in a single direction, and it is generally reserved for specific, prospectively justified circumstances. In exploratory phase 2 studies, two-tailed analyses provide a more rigorous assessment of efficacy [[14], [15], [16]].

Taken together, these considerations do not diminish the importance of these pioneering studies. Rather, they indicate that the reported findings should be viewed as early stage and hypothesis-generating.

## Interpretation of Findings: Research Implications vs Clinical Practice

The clinical development of nucleoside therapy for TK2d benefited from a large body of objective longitudinal data collected across multiple international centers and analyzed independently using multiple statistical methods and interpreted within the context of a well-characterized natural history of disease [3,12,17].

In contrast, the recently published studies evaluating dC/dT therapy in heterogenous non-TK2d MDDS subjects should be viewed as a different stage of therapeutic development. In rare disorders, such as POLG disease, where no approved disease-modifying therapies exist, uncontrolled reports of potential benefit can rapidly influence clinical practice. This places responsibility on investigators and clinicians to distinguish early expanded access observations regarding feasibility, safety, and potential biological activity rather than robust evidence of clinical efficacy. Failure to adhere to this boundary may lead to premature clinical use that outpaces necessary clinical trial evidence. Such practice may also complicate the conduct of future controlled clinical trials by reducing equipoise, limiting future subject recruitment, and impeding generation of the GCP-compliant evidence required to determine which patients truly benefit from treatment.

An additional consideration of dC/dT therapy is long-term biological safety. Although this therapy has demonstrated an acceptable safety profile in TK2d, chronic systemic excesses of pyrimidine nucleosides are pathogenic in mitochondrial neurogastrointestinal encephalomyopathy (MNGIE) [18,19]. In this severe multisystemic autosomal recessive MDDS, thymidine phosphorylase deficiency causes toxic elevations in deoxythymidine and deoxyuridine. While the clinical relevance of this observation to chronic nucleoside supplementation in non-TK2d disorders remains uncertain, nucleoside toxicity highlights the requirements for ongoing pharmacodynamic monitoring and long-term safety evaluation as these therapies are investigated in additional MDDS populations.

These issues should not be interpreted as opposition to compassionate use, expanded access, or carefully conducted N-of-1 investigations. On the contrary, these approaches remain essential components of therapeutic development in ultra-rare diseases with substantial unmet medical need. Nevertheless, their value depends on rigorous clinical evaluation, systematic outcome collection, transparent reporting of uncertainty, and continued commitment to prospective studies capable of establishing efficacy.

We hope that these considerations contribute constructively to the ongoing development of nucleoside therapy and help guide future translational and clinical research aimed at improving outcomes for patients with mitochondrial DNA maintenance disorders.

### On behalf of the TK2d collaborators who contributed to this commentary

Cristina Dominguez-Gonzalez MD PhD, Hacer Durmas MD PhD, Ayman W. El-Hattab MD, Caterina Garone MD PhD, Amy Goldstein MD, Richard Haas MD, Rita Horvath MD PhD, Thomas Klopstock MD, Carlos Lopez-Gomez PhD, Robert McFarland MA MBBS PhD, Michelangelo Mancuso MD PhD, Cristiane Moreno MD PhD, Andres Nascimento MD PhD, Vincent Procaccio MD PhD, Shamima Rahman MD PhD, Fernando Scaglia MD PhD, John L.P. Thompson PhD, Zarazuela Zolkipli-Cunningham MD.

## Author contributions

Drs. Michio Hirano, Andres Berardo, Emanuele Barca, Valentina Emmanuele, Austin Larson, Antonella Spinazzola, and Amel Karaa drafted the manuscript. All co-authors reviewed and edited the manuscript and approved the final version.

## Funding

No funding has supported this manuscript.

## Declaration of interests

The authors declare the following financial interests/personal relationships which may be considered as potential competing interests:

Michio Hirano reports a relationship with UCB that includes: consulting or advisory, funding grants, speaking and lecture fees, and travel reimbursement. Michio Hirano has patent #US Patent No.: 10,471,087 licensed to UCB. Michio Hirano serves on an advisory board of UCB; has received research support, honoraria or both from Entrada Therapeutics, Modis Therapeutics (a wholly owned subsidiary of Zogenix/UCB), Precision BioSciences, Cyclerion Therapeutics, Reneo Pharmaceutical, Sarepta Therapeutics, and Stealth BioTherapeutics; and has received grant support from the US Department of Defense (FPA W81XWH2010807), the J. Willard and Alice S. Marriott Foundation, the Muscular Dystrophy Association (577392) and the National Institutes of Health (NIH; U54 NS078059 and P01 HD32062). He also serves on the scientific and medical advisory boards of the Barth Syndrome Foundation and the United Mitochondrial Disease Foundation and is a member of the Research Advisory Committee of the Muscular Dystrophy Association.

Cristina Domínguez-González serves on an advisory board of UCB; has received funding from UCB to cover travel expenses to medical conferences and as a speaker; has received funding from UCB for research projects related to TK2d.

Ayman El-Hattab serves on the UCB TK2 Steering Committee.

Caterina Garone serves on an advisory board of UCB and has received funding from UCB for research projects related to TK2d.

Amy Goldstein is a paid consultant for UCB and serves on the UCB TK2 Steering Committee. Columbia University Irving Medical Center (CUIMC) has a patent for deoxynucleoside therapies for mitochondrial DNA depletion syndrome, including TK2d, which is licensed to Modis Therapeutics, a wholly owned subsidiary of Zogenix/UCB; this relationship is monitored by an unconflicted external academic researcher. Michio Hirano and Caterina Garone are co-inventors of this patent. CUIMC has received royalty payments related to the development and commercialization of the technology; Michio Hirano and Caterina Garone have received shares of the royalty payments following Columbia University policies.

Richard Haas has consultant agreements with Stealth BioTherapeutics, Sun Pharma Advanced Research Company and UCB. He has received research funding from the NIH North American Mitochondrial Disease Consortium (Director Career Enhancement program), Stealth BioTherapeutics, Taysha Gene Therapies and Tisento Therapeutics, and funding for research projects related to mitochondrial disease.

Thomas Klopstock is a paid consultant for UCB and serves on the UCB TK2 Steering Committee.

Austin Larson is a paid consultant for UCB and serves on the UCB TK2 Steering Committee.

Robert McFarlane is a paid consultant for UCB and serves on the UCB TK2 Steering Committee.

Andres Nascimento is a paid consultant for UCB serves on the UCB TK2 Steering Committee.

Vincent Procacio serves on the UCB TK2 Steering Committee.

Fernando Scaglia serves on advisory boards of Nestlé, UCB and Zevra Therapeutics (formerly Acer Therapeutics); has consultant agreements with Precision BioSciences and Tisento Therapeutics; has received research support from Astellas Pharma, the NIH (U54 NS078059 and U54 NS115198), PTC Therapeutics, Saol Therapeutics, Stealth BioTherapeutics and the US Food and Drug Administration's Office of Orphan Products Development (5R01-FD005407). He is also the Program Chair of the Mitochondrial Medicine Society.

Zuela Zolkipli-Cunninham is a paid consultant for UCB and serves on the UCB TK2 Steering Committee.

If there are other authors, they declare that they have no known competing financial interests or personal relationships that could have appeared to influence the work reported in this paper.
